# Gli promotes epithelial-mesenchymal transition in human lung adenocarcinomas

**DOI:** 10.18632/oncotarget.11246

**Published:** 2016-08-12

**Authors:** Hui Li, Dongsheng Yue, Joy Q. Jin, Gavitt A. Woodard, Bhairavi Tolani, Thomas M. Luh, Etienne Giroux-Leprieur, Minli Mo, Zhao Chen, Juanjuan Che, Zhenfa Zhang, Yong Zhou, Lei Wang, Xishan Hao, David Jablons, Changli Wang, Biao He

**Affiliations:** ^1^ Thoracic Oncology Program, Department of Surgery, Helen Diller Family Comprehensive Cancer Center, University of California, San Francisco, CA 94115, USA; ^2^ Department of Lung Cancer, Lung Cancer Center, Tianjin Medical University Cancer Institute and Hospital, Tianjin 300060, China; ^3^ Beijing ACCB Biotech Ltd., Beijing 100084, China; ^4^ Department of Oncology, Beijing Friendship Hospital of Capital Medical University, Beijing 100050, China; ^5^ Department of Thoracic Surgery, Fourth Hospital of Hebei Medical University, Shijiazhuang, Hebei 050011, China

**Keywords:** sonic hedgehog, gli, epithelial-mesenchymal transition, lung cancer, adenocarcinoma

## Abstract

Adenocarcinoma is the most common type of lung cancer. Epithelial-mesenchymal transition (EMT) is required for tumor invasion/metastasis and the components that control this process are potential therapeutic targets. This study we examined the role of Gli in lung adenocarcinoma and whether its activation regulates metastasis through EMT in lung adenocarcinoma. We found that tumors with high Gli expression had significantly lower E-Cadherin expression in two independent cohorts of patients with lung adenocarcinoma that we studied. *In vitro* up-regulation of SHh resulted in increased cell migration while small molecule inhibitors of Smo or Gli significantly reduced cell mobility both in a wound healing assay and in a 3D cell invasion assay. Inhibition of Gli *in vivo* decreased tumor growth and induced an increase in E-Cadherin expression. Our results indicate that Gli may be critical for lung adenocarcinoma metastasis and that a novel Gli inhibitor shows promise as a therapeutic agent by preventing cell migration and invasion *in vitro* and significantly reducing tumor growth and increasing E-Cadherin expression *in vivo*.

## INTRODUCTION

Lung cancer remains the leading cause of cancer-related mortality in the United States and worldwide with an overall 5 year survival rate of 19.3% [1]. Adenocarcinoma is the most common form of lung cancer and accounts for 38% of all newly diagnosed lung cancers [2]. Recent advancements in targeted therapies such as EGFR tyrosine kinase inhibitors and ALK inhibitors have led to modest improvements in survival times for certain subgroups of patients with lung adenocarcinoma, however, additional therapeutic strategies are desperately needed [3, 4]. Once patients have developed metastatic disease only 15% will be alive after one year and there are virtually no longer term survivors [5], underlying the importance in developing treatment strategies which target the mechanisms leading to tumor invasion and metastasis.

Epithelial-mesenchymal transition (EMT) is a process in which cells lose their cell-cell adhesive properties and gain migratory and invasive potential. EMT is essential for events in embryonic development, wound healing, fibrosis, and in cancer progression and metastasis. It has been well established that epithelial cancer cells undergo EMT for tumor progression and in order to invade local tissue and metastasize [6, 7]. EMT process is regulated by various signaling pathways including Sonic Hedgehog (SHh) which lead to many complex changes in the expression and function of proteins. The loss of the cell-to-cell adhesive protein E-Cadherin, which is inactivated in many cancers, is a fundamental event in EMT. During EMT, E-Cadherin is replaced with N-Cadherin, which enhances the motility of tumor cells [8]. EMT confers on cells the critical traits required for seeding metastasis and for developing the stem cell properties that allow for launching of new cancer cell colonies [9]. Acquisition of EMT features has been associated with poor prognosis as well as resistance to chemotherapy [10–12]. Further knowledge of the process of EMT can improve our understanding of tumor recurrence and metastasis and identify potential therapeutic targets.

The role of SHh pathway in cancer development is suspected to be via the transformation of adult stem cells into cancer stem cells. Activated SHh has been implicated in tumorigenesis and metastasis in multiple types of cancers including lung, brain, breast, prostate, and skin. In the canonical SHh pathway, the absence of the SHh ligand leads the transmembrane receptor Patched (Ptch) to inhibit the transmembrane receptor Smoothened (Smo). Inhibited Smo causes cleavage of Gli to the N-terminal repressor form. Therefore when SHh binds to Ptch, the inhibitory effect on Smo is released and active full length Gli is transported into the nucleus and activates transcription of Gli-dependent target genes such as *Gli1*, *Ptch1*, *CyclinD1* and *Wnt* [13–16]. However, non-canonical Gli activation independent of SHh, has been shown in many cancer cells types [17, 18], and there is evidence for mechanisms of Gli activation independent of SHh, stimulated by other oncogenic signaling pathways such as transforming growth factor β (TGFβ), epidermal growth factor receptor (EGFR), RAS and AKT/PI3K pathways [19–23]. As Gli transcription factors constitute the final effectors of the SHh pathway, and are implicated in multiple other oncogenic signaling pathways, they represent an important downstream target for potential cancer therapeutics [17].

The relationship of SHh pathway to EMT has not been previously studied in lung adenocarcinomas and the existing data from other solid tumors is controversial. There is a growing body of literature that shows that SHh/Gli inhibition blocks EMT, however the exact mechanisms remain to be elucidated. Some studies in melanoma and pancreatic cancers have suggested that Gli facilitates cancer cell migration and invasion via E-Cadherin [24, 25]. In lung squamous cell cancer (SCC) and in hepatocellular carcinoma, Gli expression has been shown to be inversely correlated with E-Cadherin expression and in lung SCC inhibition of the SHh pathway increases E-Cadherin expression [26, 27]. In hepatocellular cancer, Gli1 over-expression is correlated with capsular invasion, advanced tumor stage, vascular invasion and intrahepatic metastasis and interfering with Gli transcription suppresses cell migration by down-regulating matrix metalloprotease (MMP)-2 and MMP-9 [28]. *In vitro* down-regulation of Gli1 with siRNA reduced hepatoceullular cancer cell invasion and increased E-Cadherin expression [27]. However there is some conflicting data that showed inhibition of Gli promoted EMT in pancreatic cancer [29]. We have recently demonstrated increased SHh signaling in lung SCC and that Gli1 expression is inversely correlated with the EMT marker E-Cadherin. Inhibition of the SHh pathway up-regulates E-Cadherin expression and suppresses lung SCC cell migration [26].

Here, we report the Gli activation in two cohorts of patients with lung adenocarcinomas and show that Gli1 and EMT markers are inversely correlated in lung adenocarcinoma. Inhibition of Gli suppresses migration of lung adenocarcinoma cells and up-regulates E-Cadherin expression *in vitro*. We also demonstrate a reduction in lung adenocarcinoma growth *in vivo* by a small molecule Gli inhibitor.

## RESULTS

### Gli expression inversely correlates with E-Cadherin expression in lung adenocarcinoma

**Figure 1:**
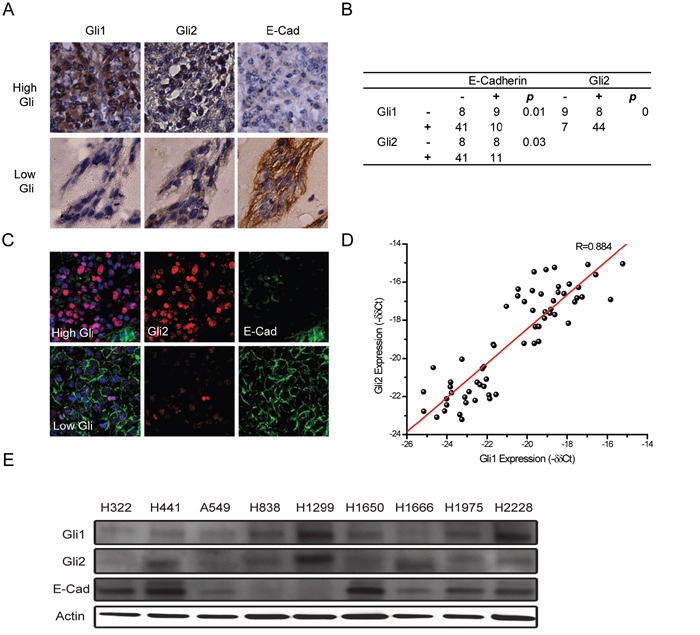
Gli expression inversely correlates with E-Cadherin expression in lung adenocarcinoma **A.** Expression of Gli1, Gli2 and E-Cadherin (E-Cad) in two representative tissue specimens assessed by immunohistochemistry in the Tianjin cohort showing high Gli expression (upper panels) and low Gli expression (lower panels). **B.** Correlation between Gli1, Gli2 and E-Cadherin in the Tianjin cohort. Statistical analyses were performed between Gli1 and E-Cad, and also Gli2 and E-Cad. **C.** Expression of Gli2 (red) and E-Cadherin (E-Cad) (green) in two representative tissue specimens was analyzed by immunofluorescence in the UCSF cohort with high Gli expression (upper panels) and low Gli expression (lower panels). DAPI (blue) was used to stain the nuclei of cells. **D.** Correlation between Gli1 and Gli2 mRNA levels by quantitative RT-PCR in the UCSF cohort. **E.** Gli1, Gli2 and E-Cad protein expression in human lung adenocarcinoma cell lines assessed by Western blotting. Actin was used as a loading control.

We investigated the expression of Gli proteins and E-Cadherin in lung adenocarcinoma patient tissues from the Lung Cancer Center at Tianjin Medical University Cancer Institute and Hospital, Tianjin and the Thoracic Oncology Program at University of California, San Francisco. The expression of Gli1, Gli2 and E-Cadherin was evaluated by immunohistochemistry (IHC) with 68 formalin-fixed, paraffin-embedded tissue specimens from the Tianjin cohort. Clinical and demographic information from the Tianjin cohort is summarized in Table 1. Tumor samples with high Gli1 or Gli2 expression showed lower E-Cadherin expression while low Gli expression showed high expression with an epithelial growth pattern (Figure 1A). The protein expressions of Gli1, Gli2, and E-Cadherin were scored a “high” or “low” expression based on IHC as previously described [26]. Statistical analysis with Kendall's tau-b correlation tests revealed that both Gli1 and Gli2 were significantly inversely correlated with E-Cadherin expression (*p*-value 0.01 and 0.03 respectively) (Figure 1B). In addition, we found E-Cadherin exhibited a heterogeneous pattern in some tissue samples, and Gli expression seemed inversely correlated with E-Cadherin within the same specimen. To confirm the inverse correlation between Gli and E-Cadherin, we conducted co-immunofluorescence of Gli2 and E-Cad on 17 tissue samples from UCSF, and 13 of these 17 (76%) samples exhibited a clear inverse correlation between Gli2 and E-Cadherin. Four of 17 (24%) samples exhibited heterogeneous expression patterns of Gli2 and E-Cadherin. Within one representative sample (Figure 1C), high Gli2 areas showed low E-Cadherin expression whereas low Gli2 areas showed high E-Cadherin expression with a clear epithelial pattern, further verifying the inverse correlation of E-Cadherin and Gli. No available combination of antibodies allowed us to perform co-staining of Gli1 and E-Cadherin; however, we demonstrated that Gli1 and Gli2 expression correlated significantly in lung adenocarcinoma by IHC on FFPE samples of the Tianjin cohort (Figure 1B) and by RT-PCR on 63 freshly frozen samples of the UCSF cohort (Figure 1D). Consistent with the tissue expression analysis, Gli expression negatively correlated with E-Cadherin expression in nine human lung adenocarcinoma cell lines assessed by Western blotting (Figure 1E). Taken together, our results demonstrated Gli expression inversely correlates with E-Cadherin expression in lung adenocarcinoma.

**Table 1:** Patient Information

Characteristics	Number	Percent
**Age (Years)**		
<60	44	64.70%
≥60	24	35.30%
**Gender**		
Male	40	85.30%
Female	28	14.70%
**Smoking history**		
Never	32	47.10%
Smoker	36	52.90%
**Surgical Procedure**		
Lobectomy	60	88.20%
Pneumonectomy	8	11.80%
**T stage**		
T1	23	7.90%
T2	39	57.40%
T3	6	8.80%
**Postoperative chemotherapy**		
Yes	44	64.70%
No	24	35.30%
**Postoperative radiotherapy**		
Yes	10	14.70%
No	58	85.30%

### SHh/Gli signaling promotes cell migration and invasion

Reduced levels of E-Cadherin have been shown to promote cancer cell mobility and invasive capability. To elucidate whether SHh/Gli signaling enhances cell migration, we carried out a wound-healing assay in two lung adenocarcinoma cell lines A549 and H1666. To inhibit SHh/Gli signaling, we applied Gli-I and Vismodegib (also known as GDC-0449). Our lab has previously developed Gli-I, a small molecule inhibitor targeting Gli, and showed it effectively inhibited cell proliferation both *in vitro* and *in vivo* by interfering Gli transcriptional activity [30, 31]. Vismodegib is a Smo inhibitor approved by the U.S. Food and Drug Administration to treat adult patients with basal cell carcinoma [32–35]. It is currently being investigated in clinical trials to treat other types of cancer due to its ability to selectively target SHh signaling [32, 36]. To stimulate the pathway, we treated cells with a recombinant SHh protein.

**Figure 2:**
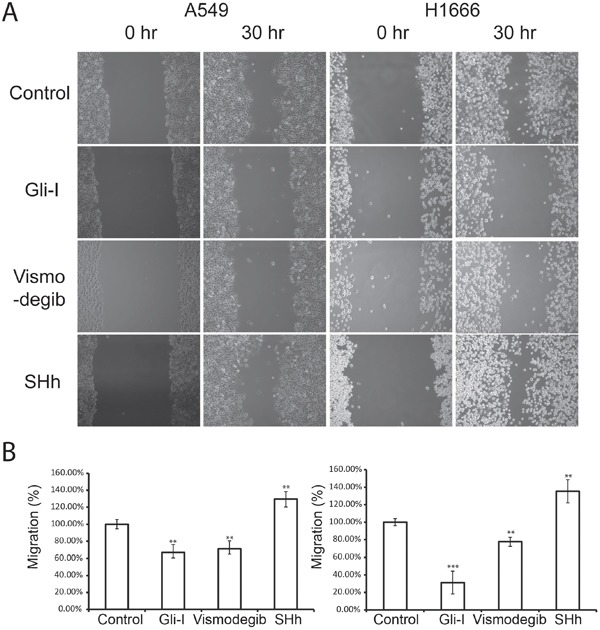
SHh/Gli signaling promotes cell migration in lung adenocarcinoma **A.** Wound healing assays of lung adenocarcinoma A549 cells (left) and H1666 (right) treated with Gli-I, Vismodegib, and recombinant SHh proteins. Representative images shown at 0 hr and 30 hr were captured using a light microscope (100X). **B.** Quantification of wound healing assays. The migration distance of cells at 0 hr was set as 100%. Statistical analysis was performed between control and treated groups. *p* values of <0.05 <0.01 or <0.001 are indicated as *, ** or *** respectively.

**Figure 3:**
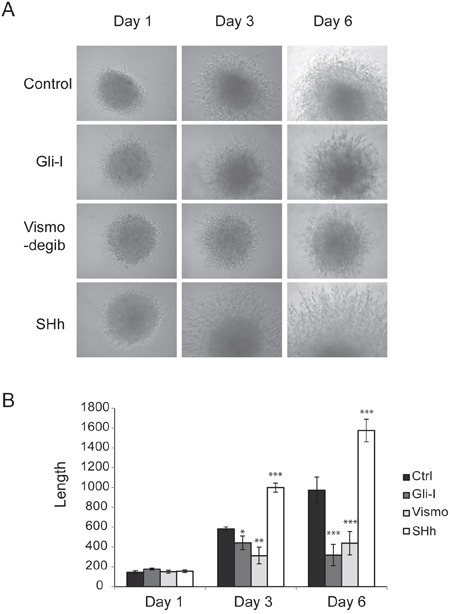
SHh/Gli signaling enhances cell invasion **A.** 3D spheroid cell invasion assay of lung adenocarcinoma A549 cells treated with Gli-I, Vismodegib, and recombinant SHh proteins. Representative images shown at day 1, day 3 and day 6 were captured using a light microscope (100X). **B.** Quantification of 3D spheroid cell invasion assays. Quantification was carried out by measuring the distance between the invasive cell frontier and spheroid edge. Statistical analysis was performed between control and treated groups. *p*-values of <0.05 <0.01 or <0.001 were indicated as *, ** or *** respectively.

Our results illustrated that down-regulation of SHh/Gli at different points in the signaling pathway with either Gli-I or Vismodegib reduced cell mobility significantly in both cell lines, while up-regulation of the pathway enhanced cell migration. Addition of Gli-I significantly reduced cell migration in A549 (*p*<0.01) and in H1666 (*p*<0.001). Vismodegib similarly reduced cell migration in A549 (*p*<0.01) and in H1666 (*p*<0.05) (Figure 2). To evaluate if SHh/Gli signaling promotes cell invasiveness, we performed *in vitro* Matrigel 3D invasion assays on A549 with Gli-I, Vismodegib and SHh treatment, and observed cell invasion on days 1, 3 and 6. The inhibition of SHh/Gli signaling significantly suppressed the invasive capability of cells, while SHh stimuli induced dramatic cell invasion. Quantification was carried out by measuring the distance between the invasive cell frontier and spheroid edge. The addition of SHh recombinant proteins significantly increased cell invasion at Day 3 (*p*<0.001) and Day 6 (*p*<.001). In contrast, use of the Smo inhibitor, Vismodegib significantly reduced cell invasion on Day 3 (*p*<0.01) and Day 6 (*p*<0.001) and similarly use of a novel Gli inhibitor significantly reduced cell invasion on Day 3 (*p*<0.05) and Day 6 (*p*<0.001) (Figure 3). Our results strongly suggest that SHh/Gli signaling promotes cell migration and invasion in lung adenocarcinoma and that inhibitors of this pathway that target Gli or Smo significantly suppress this invasion *in vitro*.

### Inhibition of SHh/Gli signaling restores E-Cadherin expression *in vitro*

**Figure 4:**
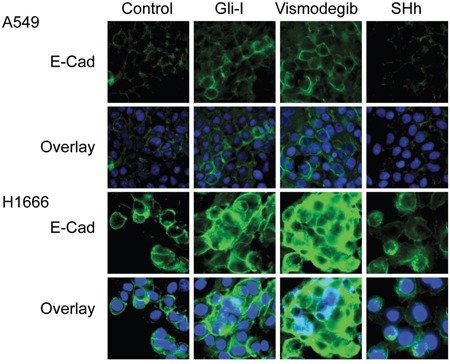
SHh/Gli signaling reduces E-Cadherin expression Immunofluorescent staining of E-Cadherin (green) in lung adenocarcinoma A549 and H1666 cells treated with Gli-I, Vismodegib, and recombinant SHh proteins. DAPI (blue) was used to stain the nuclei cells.

To determine whether E-Cadherin expression is regulated by the SHh/Gli pathway, we examined E-Cadherin expression upon Gli-I, Vismodegib and SHh treatment in cell lines A549 and H1666. E-Cadherin expression was restored and enhanced upon SHh inhibition with Vismodegib or the Gli inhibitor, while E-Cadherin expression was decreased upon SHh pathway stimulation with SHh recombinant proteins (Figure 4).

### *In vivo* inhibition of SHh/Gli reduces tumor growth and restores E-Cadherin expression

**Figure 5:**
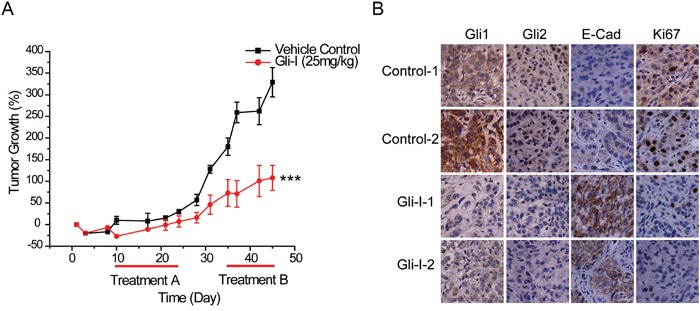
Inhibition of SHh/Gli signaling restores E-Cadherin expression *in vivo* **A.** A549 xenograft tumor growth post Gli-I treatment. Treatment was performed from day 10 for 14 days (Treatment A), followed by a 10-day interval, and a second 10-day treatment (Treatment B). Tumor size was measured every 3-4 days, and was calculated by using the equation x^2^y (where x<y), and presented as percentage of its initial volume on day 1. Statistical analysis was performed between control and the treated group at the end of the treatment. A *p* value of <0.001 was indicated as ***. **B.** Gli1, Gli2, E-Cad and Ki67 expression in the resected xenograft tumor assessed by immunohistochemistry in two representative samples in control and treated groups respectively. Mice treated with Gli-I show decreased Gli expression, increased E-Cadherin expression, and decreased Ki67 expression.

We further examined whether inhibition of SHh/Gli signaling could restore E-Cadherin expression, by conducting *in vivo* analyses in a xenograft lung tumor model of A549 cells. Ten days after implantation of A549 cells, mice received daily intraperitoneal injections of Gli-I at 25 mg/kg for 14 days, and another round of treatment for 10 days after an interval of 10 days. Gli-I treatment significantly inhibited tumor growth by 69% (*p*<0.001) at the end of the second round of treatment compared with the control group (Figure 5A). Neither an obvious change of body weight nor any noticeable toxicity in major organs form the treated mice were observed during the course of the treatments (data not shown). We examined the resected xenograft tumor specimens after the completion of the *in vivo* experiment by immunohistochemistry, and two representative samples from each group are shown in Figure 5B. Gli1 and Gli2 proteins were down-regulated in Gli-I-treated tumors compared with the control group. A decrease in Ki-67 levels confirmed the reduction of proliferation in Gli-I-treated tumors. Consistent with our *in vitro* data in cultured cells, E-Cadherin expression was induced, and then restored with a typical cell-cell junction pattern (Figure 5B).

Our results demonstrate that inhibition of SHh/Gli signaling have the capability to restore E-Cadherin expression both *in vitro* and *in vivo*, indicating that SHh/Gli signaling regulates EMT in lung adenocarcinoma through suppression of E-Cadherin.

## DISCUSSION

Our study is the first to demonstrate that activation of the SHh/Gli pathway is associated with changes in expression of EMT markers in lung adenocarcinoma. We are also the first to show the effect of SHh/Gli pathway on inhibition of lung adenocarcinoma cell migration *in vitro* and reduction of tumor growth using a Gli inhibitor *in vitro*.

The SHh/Gli pathway is known to play an important role in many solid malignancies. The downstream effects of the SHh pathway are involved with EMT and the cellular players that orchestrate the process are potential drug targets to prevent cell invasion and metastasis. Vismodegib is a Smo inhibitor that prevents the Smo transmembrane protein from transducing the SHh signal to the nucleus, thereby preventing the activation of SHh target genes. Vismodegib is approved for clinical use in patients with basal cell carcinoma and is in trials to expand its use to other cancers [32, 36]. Inhibition of Gli may be more effective than inhibition of Smo in preventing downstream SHh gene activation given evidence for non-canonical Gli activation independent of SHh in cancer cells and evidence that Gli is a target of other oncogenic signaling pathways [17–22]. Here we provide strong preliminary evidence that *in vitro* and *in vivo* Gli inhibition impairs the migratory and invasive properties of lung adenocarcinoma cells by inhibition of EMT.

In this study we show that lung adenocarcinoma specimens from two cohorts of patients, Gli1 and Gli2 are inversely correlated with the cell adhesions protein E-Cadherin, suggesting that high levels of Gli are associated with markers of EMT. We further demonstrate that the addition of either a Smo inhibitor or a Gli inhibitor significantly reduced migration of lung adenocarcinoma cells *in vitro*, whereas the addition of SHh ligand significantly increased their migration. These findings are reproduced in a 3D cell invasion simulation assay. To elucidate potential mechanisms responsible for these declines in cell migration and invasion with inhibition of the SHh pathway, we examined expression levels of the EMT marker E-Cadherin. When the SHh pathway was inhibited with Smo or Gli inhibitors E-Cadherin expression was enhanced and when the SHh pathway was stimulated with SHh recombinant proteins, E-Cadherin expression declined.

The regulation of EMT is a sophisticated process. We speculate that SHh signaling might regulate EMT at different levels. It has been suggested that the transcriptional targets of SHh signaling includes key genes that are involved in the EMT process, such as snail1 [37, 38]. The cross-talks between SHh and TGF-beta signaling may play an important role in the regulation [37, 38]. Besides, the possible protein-level interaction between Gli proteins and beta-catenin at cell-cell junctions might also contribute to the regulation [39]. The molecular mechanism how the SHh pathway regulates EMT in lung adenocarcinoma remains to be further explored.

Most importantly, we demonstrated the successful use of a Gli inhibitor *in vivo* to suppress the growth of lung adenocarcinoma in a xenograft model without any toxicity and in these tumors showed histologically that Gli inhibition led to decreased Gli expression, increased E-Cadherin, and decreased markers of cellular proliferation.

While Vismodegib has shown promise at reducing tumor size and lengthening progression free survival times [34], resistance mechanisms have already been identified. A *Smo* mutation, D477G, that prevents Vismodegib from binding to Smo was identified in a medulloblastoma patient who had relapsed after an initial response [40], and another *Smo* mutation, E518K, has also been identified in humans [41]. Research is underway into overcoming these resistance mechanisms with second generation Smo inhibitors such as Itraconazole, which interacts with Smo at a different binding site, and arsenic trioxide which promotes the degradation of Gli2 transcription factor before it can translocation to the nucleus and affect gene expression. Itraconazole and arsenic trioxide both inhibit the growth of medulloblastoma and basal cell carcinoma *in vitro* and *in vivo*, and prolong survival of mice with intracranial drug-resistant Smo [42]. Inhibiting the SHh pathway with a Gli inhibitor is favored due to avoidance of potential upstream resistance mechanisms that have posed a challenge to the currently approved Smo inhibitor. In addition, direct Gli inhibition can block both canonical and non-canonical activation of Gli, thereby preventing gene transcription that is a target of multiple oncogenic pathways. While further research is still required, Gli inhibition is a promising area for further investigation and potential drug development.

## MATERIALS AND METHODS

### Tissue specimens

Tissue specimens of the UCSF cohort were collected from 17 patients who underwent surgical resection for lung adenocarcinoma at the Thoracic Oncology Program at UCSF. Tissue specimens of the Tianjin cohort were collected from 68 patients who underwent surgical resection for lung adenocarcinoma at the Tianjin Medical University Cancer Institute and Hospital. Samples were fixed in formalin and embedded in paraffin to make tissue slides. The study with UCSF patient tissues was approved by the Committee on Human Research (CHR approval number: H8714-11647-10) at the University of California, San Francisco (UCSF), and that with Tianjin cohort was approved by the Tianjin Medical University Cancer Institute and Hospital. Written, informed consent was obtained from each patient before specimen collection.

### Immunohistochemistry, immunofluorescence and western blot

Immunohistochemistry (IHC), immunofluorescence (IF) and western blot were performed following standard procedures. Antibodies applied to detect protein expressions in IHC and IF were Gli1 1:100 (sc-20687 Santa Cruz Biotechnology, Santa Cruz, CA), Gli2 1:150 (Abcam, Cambridge, MA), E-Cadherin 1:100 (EMD Millipore, Billerica, MA), and the cell proliferation marker Ki67 1:200 (Abcam, Cambridge, MA). Antibodies used in Western blot were Gli1 1:100 (sc-20687 Santa Cruz Biotechnology), Gli2 1:150 (Abcam, Cambridge, MA), E-Cadherin 1:100 (EMD Millipore), and Actin 1:500 (Sigma-Aldrich, St. Louis, MO). Total protein extraction was performed with M-PER Mammalian Protein Extraction Solution (Thermo Scientific, Waltham, MA), and 40 μg of protein extracts were analyzed by Western blotting.

### RNA extraction and RT-PCR

Total RNA was isolated from tissue or cultured cells using an RNeasy kit (Qiagen, Germany). Genomic DNA contamination was eliminated by DNase I treatment. Reverse transcription was conducted with 250 ng RNA using the iScript cDNA synthesis kit (Bio-Rad, Herculas, CA). The resulting cDNAs were analyzed with real-time RT-PCR using Gene Expression Assays in a 7900 Real-Time PCR System (Applied Biosystems, Foster City, CA) for 40 cycles (96°C for 15 seconds and 60°C for 1 minute). Gene expression was normalized to 18S expression. Experiments were performed in triplicate and three times.

### Cell culture, drug treatment, migration assay and 3D culture

Human lung adenocarcinoma cell lines H322, H441, H838, H1299, H1650, H1666, H1975, and H2228 were purchased from American Type Culture Collection (Manassas, VA). The cell lines were cultured in RPMI 1640 (Life Technologies, Carlsbad, CA) supplemented with 10% fetal bovine serum (FBS) and antibiotics. Cells were seeded one day before treatment with Gli-I and Vismodegib (Selleck) at different concentrations and SHh recombinant proteins (eBioscience) were incubated for 30 hours, with vehicle (DMSO) as controls. Cells were then subjected to the following analyses of immunofluorescence, migration assay and Cultrex^®^ 3D Spheroid Cell Invasion Assay (Trevigen, Gaithersburg, MD). In migration assays, four wounds were made in each condition, and cell migration was presented by the average of distance differences between 30 hours and 0 hours. In 3D Spheroid Cell Invasion Assay, distances between the invasive cell frontier and spheroid edge were measured on day 1, day 3 and day 6. In order to observe the restoration of E-Cadherin, the cells were incubated at different conditions for 24 hours before immunofluorescence. All experiments have been conducted more than three times, and representative results were included in the text.

### Animal studies

Nude mice were subcutaneously injected with 10 million A549 cells with BD Matrigel Matrix (BD Biosciences, San Jose, CA). Ten days after inoculation, mice were randomized and intraperitoneally injected with either Gli-I at 25 mg/kg (n=10) or vehicle alone (n=10) for 14 consecutive days, followed by a 10-day observation and a 10-day Gli-I treatment regimen. Tumor volumes were measured every 3-4 days from day 1 to day 44 post-inoculation. Growth was calculated using the equation x^2^y (where x and y were the largest width and length dimensions and x<y), and presented as a percentage of the initial volume on day 1. Tumors and organs were dissected on day 44 to make paraffin blocks for follow-up IHC analyses. All experiments were performed at the University of California, San Francisco Preclinical Core. The experimental protocol was approved by and performed in accordance with guidelines from the Institutional Animal Care and Use Committee at University of California, San Francisco.

### Statistical analysis

A Kappa test was used for correlation analysis between the expressions of SHh pathway components Gli1 and Gli2 and EMT maker E-Cadherin. IHC scores of 1-3 were grouped as positive “+”, and 0 was grouped as “-“for dichotomous analysis. Two-sided student's *t*-test was performed for proliferation assays, migration assays, and 3D spheroid cell invasion assays analyses. A *p* value <0.05 was indicated as *, 0.01 as **, and 0.001 as *** in corresponding figures. Data analysis was performed using SPSS 17.0 software.
